# Molecular Docking and Simulation Studies of Antidiabetic Agents Devised from Hypoglycemic Polypeptide-P of *Momordica charantia*

**DOI:** 10.1155/2021/5561129

**Published:** 2021-09-17

**Authors:** Rawaba Arif, Sajjad Ahmad, Ghulam Mustafa, Hafiza Salaha Mahrosh, Muhammad Ali, Muhammad Tahir ul Qamar, Hafiza Rabia Dar

**Affiliations:** ^1^Department of Biochemistry, Government College University, Faisalabad 38000, Pakistan; ^2^Department of Health and Biological Sciences, Abasyn University, Peshawar, Pakistan; ^3^Department of Biotechnology, Akhuwat Faisalabad Institute of Research Science and Technology, Faisalabad 38000, Pakistan; ^4^College of Life Science and Technology, Guangxi University, Nanning, China; ^5^Department of Biochemistry, University of Agriculture, Faisalabad 38040, Pakistan

## Abstract

Diabetes mellitus termed as metabolic disorder is a collection of interlinked diseases and mainly body's inability to manage glucose level which leads to cardiovascular diseases, renal failure, neurological disorders, and many others. The drugs contemporarily used for diabetes have many inevitable side effects, and many of them have become less responsive to this multifactorial disorder. *Momordica charantia* commonly known as bitter gourd has many bioactive compounds with antidiabetic properties. The current study was designed to use computational methods to discover the best antidiabetic peptides devised from hypoglycemic polypeptide-P of *M. charantia*. The binding affinity and interaction patterns of peptides were evaluated against four receptor proteins (i.e., as agonists of insulin receptor and inhibitors of sodium-glucose cotransporter 1, dipeptidyl peptidase-IV, and glucose transporter 2) using molecular docking approach. A total of thirty-seven peptides were docked against these receptors. Out of which, top five peptides against each receptor were shortlisted based on their S-scores and binding affinities. Finally, the eight best ligands (i.e., LIVA, TSEP, EKAI, LKHA, EALF, VAEK, DFGAS, and EPGGGG) were selected as these ligands strictly followed Lipinski's rule of five and exhibited good ADMET profiling. One peptide EPGGGG showed activity towards insulin and SGLT1 receptor proteins. The top complex for both these targets was subjected to 50 ns of molecular dynamics simulations and MM-GBSA binding energy test that concluded both complexes as highly stable, and the intermolecular interactions were dominated by van der Waals and electrostatic energies. Overall, the selected ligands strongly fulfilled the drug-like evaluation criterion and proved to have good antidiabetic properties.

## 1. Introduction

Diabetes mellitus (DM) is widely known as a rising multifactorial disease reaching epidemic level. It has been affecting every age group without any discrimination [1]. It has been estimated by the International Diabetes Federation that almost 415 million people were suffering from diabetes in the year 2015, and by the year 2040, this will exceed to 640 million [2]. There are two main types of diabetes (i.e., type 1 and type 2). In type I DM, the body stops producing insulin, and in type II, there is a defect in insulin secretion and its action [3]. Diabetes can be treated by healthy dietary intake, regular exercise, and use of synthetic or natural drugs and maintaining a healthy lifestyle [4]. Present therapies are effective in managing diabetes but have many side effects. Limitations of current therapies are not just escalating the diabetes prevalence but also crossing the limits of economical budget. All these effects are demanding safer, efficient, easy to administrate, and budget-friendly treatment [5]. The traditional approach of treatment involves antidiabetic compounds from different plant species, and it is catching more and more attention as natural drugs show fewer side effects compared to synthetic drugs [6]. It has been estimated that almost 1200 plant species have been identified which are comprised of compounds with promising hypoglycemic activity. Plant species with respective bioactivity have also been scrutinized to find exact lead compounds for desirable activity [7].

*Momordica charantia* is commonly known as bitter gourd. Its extract has been known for effectively lowering the blood glucose level [8]. There are many hypoglycemic compounds isolated from *M. charantia* including insulin-like peptides, vicine, polypeptide-P, alkaloids, charantin, sterol glycosides, mcIRBP, triterpenoids, cucurbutanoid compounds, flavonoids, and phenols, and all these compounds have hypoglycemic activity. Water-soluble proteins (MC1, MC6, MC6.1, MC6.2, and MC6.3 and MC2–1-5) and insulin receptor- (IR-) binding protein all have antidiabetic activity [9]. Due to antihyperglycemic activity of *M. charantia*, its consumption as a dietary source and use in traditional medicines has made it more attractive for the advanced research [10]. In a study, Elekofehinti et al. [11] have reported that *M. charantia* have many bioactive compounds such as charantin, cucurbitacin, and momordicoside D which have antidiabetic properties, and all these compounds belong to saponin class. Similarly, Shivanagoudra et al. [12] isolated 3*β*,7*β*,25-trihydroxycucurbita-5,23(E)-dien-19-al, charantal, charantoside XI, and 25*ξ*-isopropenylchole 5,6-ene-3-O-D-glucopyranoside from the fruit of *M. charantia* and used in molecular docking studies. The compounds were found to have antidiabetic properties.

DM is a complicated disease because it can be caused by defects in many organs, proteins, and enzymes [13]. Due to complex nature of this disease, one cannot rely on a single experimental model and also single treatment cannot circumvent this multifactorial disease. The experimental models are the protein receptors which are involved in the regulation of glucose throughout the body such as insulin receptor and sodium-glucose cotransporter 1 and 2 [14]. Insulin receptor (IR) is a member of the protein tyrosine kinase family and a transmembrane signaling protein. IR has many crucial regulatory activities regarding cell growth, differentiation, and metabolism. Its role in the regulation of glucose homeostasis discriminates it from other members of the family [15]. Inactivation of insulin receptor by knocking out its gene leads towards the loss of insulin secretion and glucose tolerance. The studies have clearly defined the role of IR in glucose homeostasis and showed its importance in the treatment of DM [15]. Studies also reported that an altered insulin receptor activity has been indicated in type I and type II DM [16]. Sodium-glucose cotransporter 1 (SGLT1) plays a very important role in the reabsorption of glucose by facilitated diffusion from the kidney and intestine [17]. It has been reported that alteration in SGLT1 leads towards the defects in reabsorption of glucose from the kidney and intestine. Such studies have indicated that inhibition of SGLT1 might be effective in the treatment of DM [18].

In the current study, four proteins were used as target receptors including IR, SGLT1, DPP-IV, and GLUT2 proteins. The aim of the study was to explore hypoglycemic peptides devised from polypeptide-P of *M. charantia* using in silico approaches. The selected peptides were scrutinized through Lipinski's rule of five and ADMET profiling. Finally, the ligands which fulfilled all these criteria are referred as potential antihyperglycemic agents. It is hypothesized that the selected peptides as antidiabetic agents would be better and safe alternate of currently available treatments of DM. The findings of this study would be employed as a novel approach for screening of antidiabetic drugs.

## 2. Materials and Methods

Molecular docking has accelerated the drug discovery by providing the structure-based interactions between ligand and receptor protein. A total of thirty-seven peptides from polypeptide-P of *M. charantia* were prepared including tetra-, penta-, and hexapeptides. These receptor proteins were selected based on their key roles in DM and in the maintenance of glucose homeostatic. This study involves the docking of thirty-seven peptides devised from polypeptide-P of *M. charantia* against IR as agonists and against SGLT1, DPP-IV, and GLUT2 as inhibitors. The docking analysis was performed using Molecular Operating Environment (MOE) software [19].

### 2.1. Retrieval of 3D Structures of Receptor Proteins

The three-dimensional (3D) structures of IR (PDB ID: 1IR3) [20], SGLT1 (PDB ID: 3DH4) [21], and human dipeptidyl peptidase-IV (PDB ID: 4A5S) [22] as receptor proteins were retrieved from the RCSB Protein Data Bank (https://www.rcsb.org/) in .pdb format [23] while the 3D structure of GLUT2 which was predicted in our previous study [24] was also used as a target protein.

### 2.2. Refinement of Receptor Proteins

Protein structures of receptor proteins were refined using MOE software before docking studies. The receptor proteins were prepared and optimized by removing ligands and water molecules. The receptor proteins were also energy minimized, and 3D protonation was done using parameter force field gradient: 0.05.

### 2.3. Ligand Selection and Database Preparation

The protein sequence of polypeptide-P from *M. charantia* was retrieved from NCBI's Entrez Protein under accession No. ADO14327.1. Tetra-, penta-, and hexapeptides were devised from the polypeptide-P, and their 3D structures were prepared using the ChemSketch software in MOL format [25]. The peptides were energy minimized using MOE.

### 2.4. Molecular Docking

Molecular docking analysis was carried out to identify the best agonists of IR and inhibitors of SGLT1, DPP-IV, and GLUT2. The site finder tool of MOE was used to predict the active sites of both receptor proteins. The docking analysis was done using default parameters (i.e., rescoring 1: London dG; retain: 10; refinement: force field; rescoring 1: London dG; retain: 10). The most appropriate protein-ligand interactions were selected on the basis of best S-scores and binding interactions. MOE docking algorithm poses peptides within the catalytic pocket of the receptor protein with various orientations and conformational degrees of freedom. The docking analysis predicts suitable structural interactions of ligand and receptor protein based on combined scoring functions (i.e., ligand shape, electrostatic compatibility with the target, solvation effects, binding energies, and enthalpy and entropic effects, most stable binding mode with minimum energy, number of hydrogen bonds, values of root mean square deviation (RMSD), and S-scores) [26].

### 2.5. Molecular Dynamics Simulations

MD simulations were conducted using the AMBER20 suite of molecular dynamics program with the force filed FF14SB [27, 28]. AMBER force field (GAFF) using the Antechamber program was used to generate force-field parameters for the ligand molecules [29, 30]. The studied systems were then solvated by immersing each complex in a cubic box of TIP3P water molecules with a 10 Å solute-wall distance. The net charge on the complex protein was neutralized by the addition of Na^+^ ions. The energies of the solvated systems were minimized before undergoing molecular dynamics simulations. All the systems were subjected to 1,500 steps of the steepest descent algorithm and 1,000 steps of the conjugate gradient algorithm with a nonbonded cutoff of 8 Å. Standard MD simulation protocol was followed consisting of an initial heating period of 100 ps starting slowly from 0 K to a temperature of 300 K and pressure of 1 atm. Following the heating procedure, each complex system underwent 100 ps equilibration at a constant temperature of 300 K. Equilibration was followed by a production run of 5 ns for the undocked protein and 12 ns each for all the docked complexes. The Ewald summation method was applied to treat long-range electrostatic interactions. PTRAJ module of AMBER was utilized to generate output files for analysis [31]. The conformational entropy evaluation (normal mode analysis) requires large amounts of CPU resources. Therefore, the approximation of the calculation of the binding free energy by removing this term from the MM-GBSA equation has been widely used, including in this study, as the removal of the entropic evaluation can be considered for the analysis and comparison of structurally similar compounds. All the trajectories were used for the calculation. The Molecular Mechanics Generalized Born Surface Area (MM-GBSA) method [32] with the MMPBSA.py script was used to calculate the complex binding energy [33]. The MM-GBSA energy values of the 50 ns period were calculated from the representative 100 frames. All structures were visualized by UCSF Chimera [34]. The solvent-accessible surface area (SASA) was performed using a visual molecular dynamics tool [35].

### 2.6. Drug Scan and ADMET Profiling

The best selected ligands must fulfill the criteria of drug scan through Lipinski's rule of five (Ro5) [36]. The peptides selected on the basis of Ro5 are considered safe. admetSAR is an online available webserver. It is used to check ADMET-related properties of selected ligands including pharmacokinetics of drugs in the human body including absorption, distribution, metabolism, excretion, and toxicity. Ligands that accomplished all these parameters are accepted as potential drug candidates [37].

## 3. Results

The docking analysis was carried by the MOE algorithm using devised peptides against IR, SGLT1, DPP-IV, and GLUT2. The top five peptides were selected in each analysis based on their interaction patterns and energy validations.

### 3.1. Interaction Analysis

**Figure 1:**
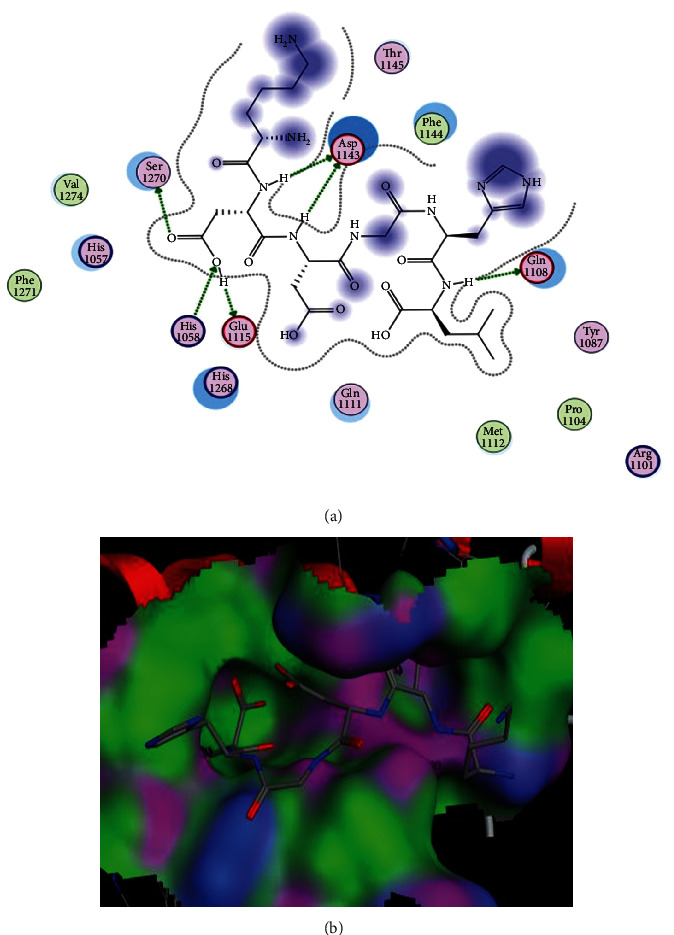
Docking of KDDGHL peptide with insulin receptor. (a) Interactions of peptide with IR. In these interactions, His1058 is a basic amino acid and acting as a sidechain donor. Glu1108, Glu1115, and Asp1143 are acidic amino acids and acting as sidechain acceptors. (b) Binding pattern of KDDGHL with the binding pocket of IR.

For insulin receptor, the top five ligands were selected based on their S-scores and interaction patterns. Results have pointed out that all the five ligands have the efficiency to bind with IR. Amino acids that were unanimous to interact with at least three peptides were referred as interacting amino acids. The peptide KDDGHL showed the best interactions (binding score: -18.56) with the IR receptor, and Ser1270, Asp1143, Glu1108, Glu1115, and His1058 were found to be the leading interactive residues in these interactions (Figure 1(a)). The binding mode of the KDDGHL peptide within the binding pocket of IR is shown in Figure 1(b). Chaetochromin was also docked against IR as a positive control because chaetochromin has been reported for its antidiabetic activity [38]. The chaetochromin showed interactions with Arg*B*1061, Ser*A*151, and Cys*B*1056. The interactions and binding pattern of chaetochromin have been shown in Figure S1. The chaetochromin showed an S-score of -19.11 and RMSD of 1.89 which is comparable with the best selected peptide in this study.

The S-scores, RMSD values, and interacting amino acids of the top five peptides with IR are given in Table 1. All selected ligands showed strong interactions with Ser1270, Asp1143, Glu1108, Glu1115, His1057, Thr1345, and Thr1145. These interactions play a vital role in the determination of stability of the ligand-receptor complex in terms of hydrogen bond and hydrophobic and electrostatic interactions. All these interacting amino acids are present in the catalytic site of the receptor protein. Following KDDGHL, the interactions and binding patterns of the next three best peptides have been shown in Figures S2 to S4 of the Supplementary file.

**Figure 2:**
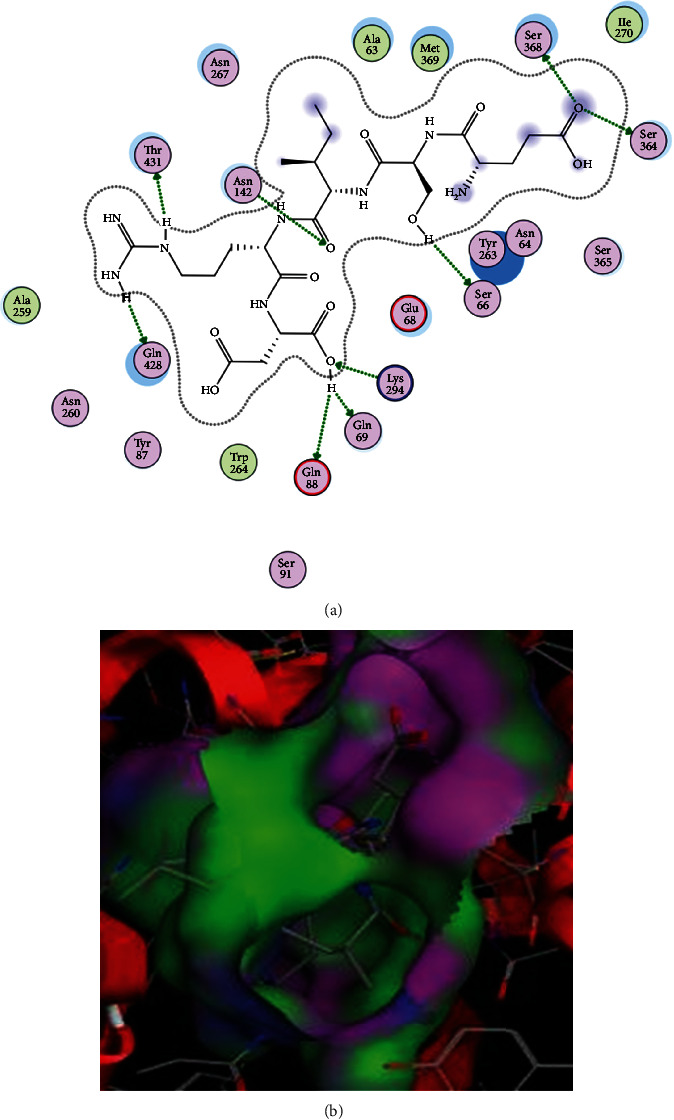
Docking of ESIRD peptide with SGLT1 receptor. (a) Interactions of peptide with SGLT1. In these interactions, Ser66, Gln69, Ser364, Ser368, Gln428, and Thr431 are polar amino acids and acting as sidechain acceptors. Asn142 is also a polar amino acid but acting as a sidechain donor. Lys294 and Glu88 are basic and acidic amino acids and acting as sidechain donor and acceptor, respectively. (b) Binding pattern of ESIRD with the binding pocket of SGLT1.

The binding pocket of SGLT1 receptor protein contains Asn267, Tyr138, Tyr263, Ser368, and Thr431 as main interacting amino acids. In the current study, out of 37 docked ligands, the top five ligands with the best S-scores and interactions with these active amino acids are given in Table 1. The peptide ESIRD showed best interactions (binding score: -23.81) with the SGLT1 receptor, and Thr431, ser368, Gln428, Asn142, Ser364, Ser66, Lys294, Gln69, and Glu88 were found to be the leading interactive residues in these interactions (Figure 2(a)). The binding mode of the ESIRD peptide within the binding pocket of SGLT1 is shown in Figure 2(b). The other four peptides DSRHR, RRKKV, and PTRHM with docking scores of -23.64, -20.64, and -19.60 showed best interactions with active amino acids of the binding pocket of SGLT1 (Figures S5-S7 of the Supplementary file). Phlorizin has been reported as an inhibitor of SGLT1 [39] and therefore selected as a positive control in this study. The peptides reported in this study showed better S-scores compared to the reported drug phlorizin (i.e., S-score of -18.25, RMSD of 1.42). The phlorizin showed interactions with amino acids Asn267, Ser68, Tyr263, Gln69, and Asn142. The interactions and binding patterns of phlorizin with SGLT1 have been shown in Figure S8 of the Supplementary file.

Similarly, the library of devised peptides was also docked against DPP-IV and GLUT2 receptor proteins. The peptide PTRHM interacted with Gln268, Thr375, and Asn371 amino acids of DPP-IV with an S-score of -10.11 (Figure S9). The interactions and binding patterns of the other two peptides, i.e., RRKKV and KDDGHL with DPP-IV, have been shown in Figures S10 and S11. The peptide RRKKV interacted with Met174, Glu361, and Arg432 amino acids of GLUT2 receptor protein and exhibited the S-score of -10.60 (Figure S12). The interactions and binding patterns of the other two peptides, i.e., RSIHEP and ERFDSG with GLUT2, have been shown in Figures S13 and S14. The detail of interactions (i.e., interacting amino acids, S-scores, and RMSD values) of the top five peptides against DPP-IV and GLUT2 as receptor proteins has been given in Table 1.

**Figure 3:**
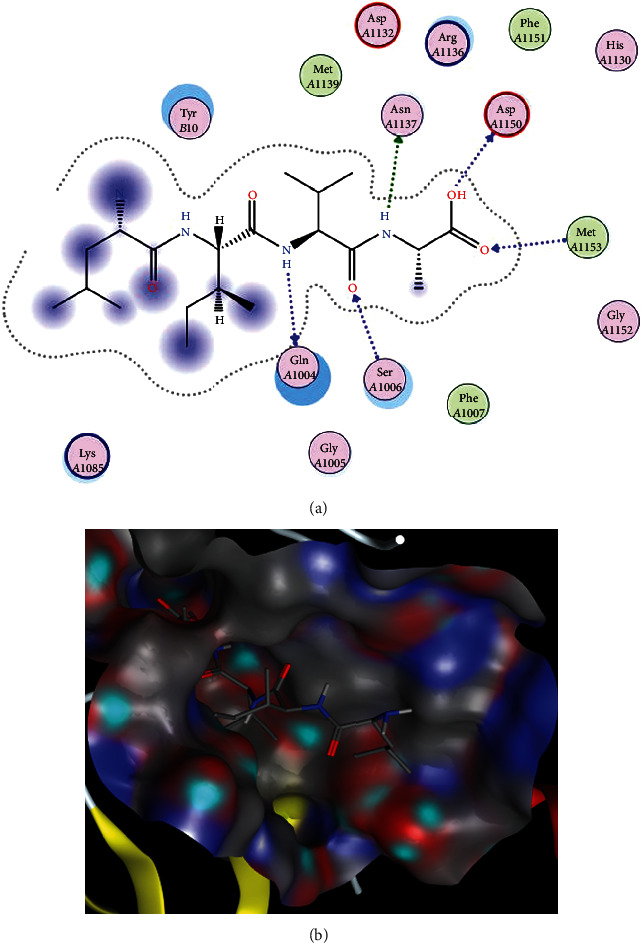
Docking of LIVA peptide with insulin receptor. (a) Interactions of LIVA with IR. In these interactions, Gln*A*1004 and Ser*A*1006 are polar amino acids and acting as backbone acceptor and backbone donor, respectively. Asn*A*1137 is a polar amino acid and acting as a sidechain acceptor. The amino acid Asp*A*1150 is acidic in nature and acting as backbone acceptor while Met*A*1153 (greasy in nature) is acting as backbone donor. (b) Binding pattern of LIVA with the binding pocket of IR.

**Figure 4:**
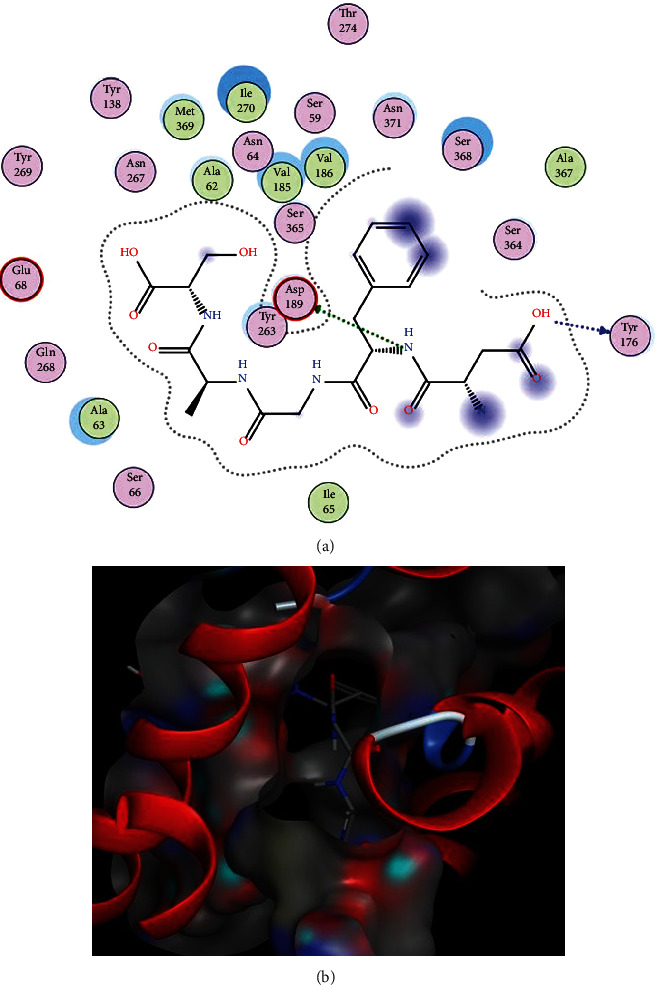
Docking of DFGAS peptide with SGLT1. (a) Interactions of DFGAS with SGLT1. In these interactions, the amino acids Tyr176 and Asp189 are polar and acidic amino acids and acting as backbone and sidechain acceptors, respectively. (b) Binding pattern of DFGAS with SGLT1.

The interactions and binding patterns of peptides LIVA with IR and DFGAS with SGLT1 receptor proteins are shown in Figures 3 and 4, respectively. Both peptides were found best among all selected peptides on the basis of their pharmacokinetic parameters which are important for the bioavailability of compounds as drugs.

### 3.2. Molecular Dynamics Simulation Study

**Figure 5:**
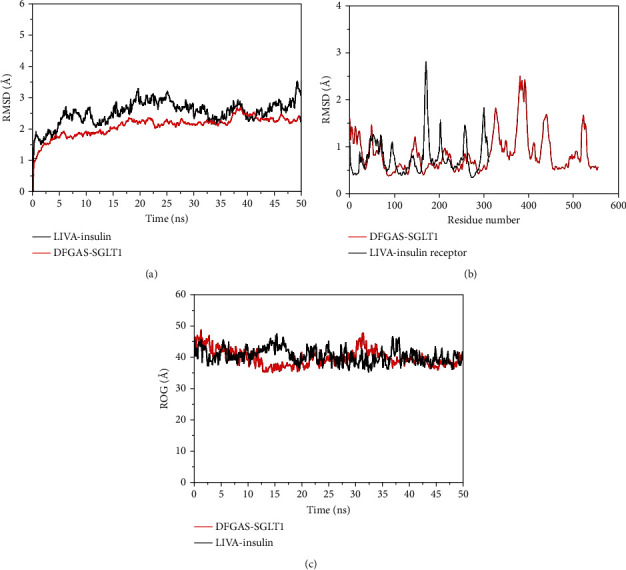
Structural stability evaluation of complexes based on carbon alpha atoms. (a) RMSD, (b) RMSF, and (c) RoG. The residue numbering is adjusted from 1 to the end.

The dynamics stability of the receptors (i.e., IR and SGLT1) in the presence of the filtered peptides was investigated by running 50 ns of molecular dynamics simulations. The structural stability of receptors was evaluated first by calculating root mean square deviation (RMSD) analysis, which computes structural deviations by overlapping simulation snapshots over docking reference snapshot based on carbon alpha atoms. Both complexes were revealed stable as the RMSD was in good acceptable range (Figure 5(a)). The RMSD plot of both complexes was found very consistent, and no major deviations were reported. The maximum RMSD touched by DFGAS-SGLT1 is around 2.2 or 2.3 Å whereas for LIVA-Insulin receptor, the maximum RMSD noticed is 3 Å. Next, root mean square fluctuations (RMSFs) were calculated for the docked receptors. Consistent with the RMSD analysis, RMSF results indicated the stable nature of complexes (Figure 5(b)). The RMSF for complexes is within 3 Å though some residues fluctuation was observed. The RMSFs correspond to the receptor loop regions and have no significant impact on the peptide bonding. Also, radius of gyration (RoG) analysis was performed to check the compactness of the complexes (Figure 5(c)). The RoG results are in coherence with the RMSD; the systems are highly compact with exception of few minor deviations. Both RoG systems fluctuated around 45 Å. The systems were seen in good equilibrium towards the end of simulation time.

Further, binding free energies of complexes were estimated as tabulated in Table 2. As can be seen in the table, both electrostatic and van der Waals energy are contributing significantly to overall system stabilization. The van der Waals seems to be playing a major role in the ligand stability at the docked site. Opposed, the solvation energy is noncontributing majorly because of polar solvation energy. The nonpolar energy term though plays a favorable part in the complex formation. Overall, both the systems acquired highly significant net binding energy values, i.e., LIVA-Insulin (-93.38 kcal/mol) and DFGAS-SGLT1 (-62.85 kcal/mol). The individual contribution of each interacting residue of the receptor to ligand was further determined by decomposing the net MM-GBSA binding free energy into residues of the receptor molecules. It was revealed that most of the residues near the binding site of the ligands contributed significantly in stabilizing the ligands as can be seen by lower binding energy value. The ligand interacting residue binding energy value is given in Table 3. The SASA analysis was performed to investigate the surface of the system accessible to the solvent (Figure S15 of the Supplementary file).

### 3.3. Drugability and ADMET Profiling

**Figure 6:**
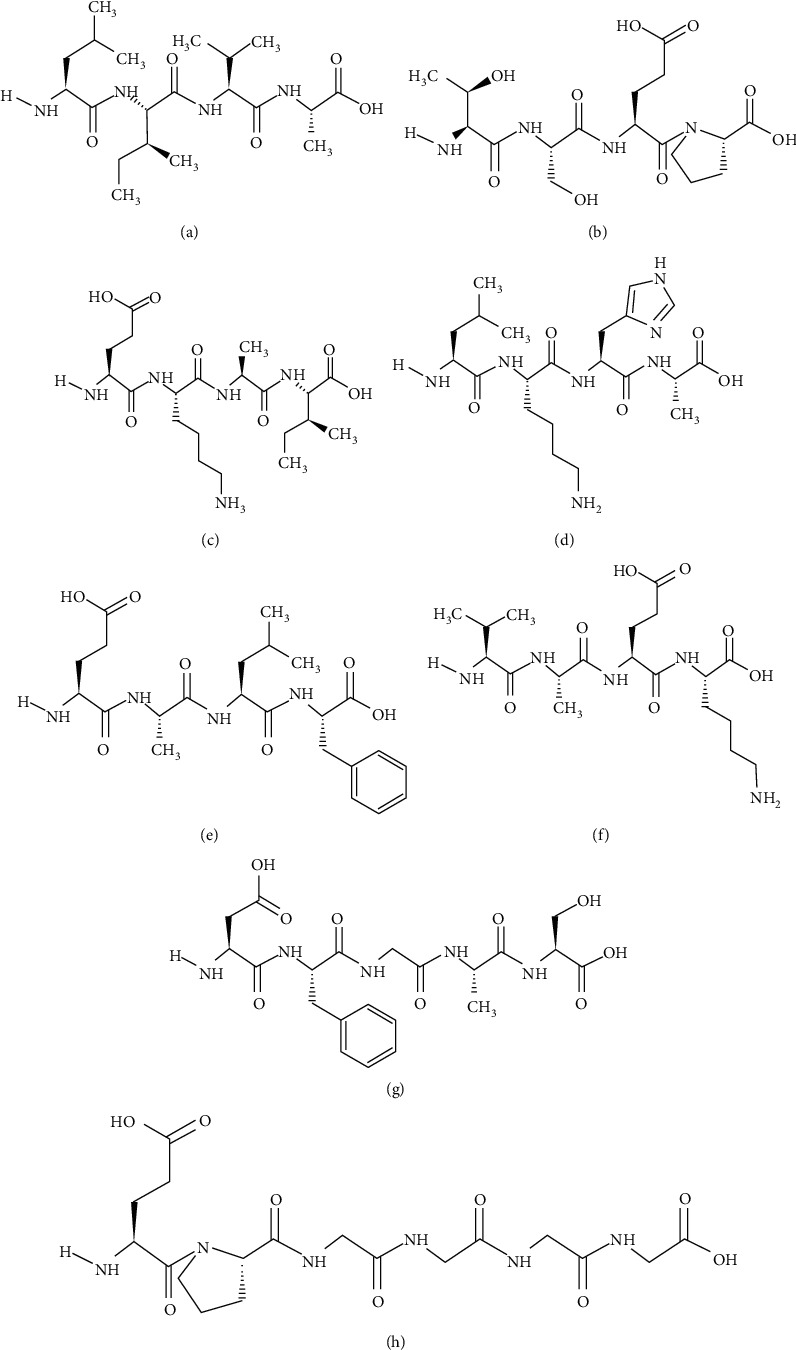
Structures of best selected peptides: (a) LIVA, (b) TSEP, (c) EKAI, (d) LKHA, (e) EALF, (f) VAEK, (g) DFGAS, and (h) EPGGGG.

Lipinski's rule of five indicates drug-like characteristics or drug potency of proposed compounds based on parameters such as molecular mass (<500 Dalton), molar refractive index (40-130), partition coefficient (Log*P* ≤ 5), hydrogen bond donors (≤5), and hydrogen bond acceptors (≤10). This rule basically distinguishes the compounds based on drug-like and non-drug-like properties. Only those compounds that fulfill these criteria are considered as good drug candidates. In this study, a total of twenty ligands were selected based on their best interaction patterns with active amino acids of the target proteins, S-scores, and energy validations. Out of twenty selected ligands, only eight peptides fulfilled the criteria of being good drug candidates according to Lipinski's Ro5 (Table 4). Six tetrapeptides, one pentapeptide, and one hexapeptide were revealed to be good agonists of IR. The hexapeptide EPGGGG has shown good binding affinity with both IR and SGLT1 receptors. The peptide (VAEK) showed good binding affinity with IR and DPP-IV. Similarly, the peptide (DFGAS) showed good binding affinity with IR, SGLT1, and DPP-IV. Out of eight, seven peptides violated only one parameter of Lipinski's rule of five but one peptide (LIVA) did not violate any rule and therefore revealed as a best potential agonist of IR. The structures of these eight peptides are given in Figure 6. On the basis of Lipinski's Ro5, these peptides could be accepted for their reasonable oral bioavailability.

The best selected peptides were further evaluated through admetSAR server to check their pharmacokinetics or ADMET (absorption, distribution, metabolism, excretion, and toxicity) profiling. The results of admetSAR have suggested that all the selected ligands are non-Ames toxic and noncarcinogens (Table 5). The evaluation of ADMET profiling of these peptides has predicted that they are tolerable and safe, and therefore, these peptides could be referred as efficient drug candidates against selected receptor proteins.

## 4. Discussion

*In silico* screening of natural compounds for drug designing has become the need of the hour due to expensive, tiresome, and laborious screening methods. A giant amount is wasted due to directionless laboratory procedures which lack structural understanding of drugs and target molecules. On the other hand, computational biology reduces the risk of late-stage failure of a drug [40]. In the current study, *M. charantia* was selected as a source of antidiabetic agent. It has been reported that a series of fractions from the fruit of *M. charantia* have been used to treat the diabetic rats. Consequently, those fractions improved the insulin signaling in diabetic rats [41]. In the current study, four target receptors were selected (i.e., IR, SGLT1, DPP-IV, and GLUT2) due to their crucial roles in maintaining the glucose level in the body [42]. Interestingly, the finding of small molecules which can elicit the IR signaling pathway and inhibit SGLT1, DPP-IV, and GLUT2 would be great alternative of insulin to treat DM.

The docking analysis of thirty-seven peptides devised from polypeptide-P of *M. charantia* was performed against IR, and the top five peptides were selected based on their scoring and binding patterns. The amino acids that were unanimously involved in structural interactions of peptides and receptors were found to be Ser1270, Asp1143, Glu1108, Glu1115, His1057, Tyr1087, and Thr1145. These amino acids are repeatedly involved in the binding interaction of each peptide with receptor protein and therefore adapted as active amino acids of the catalytic cleft. In a similar study, the antidiabetic potential of cowpea peptides was determined which can act as agonists of insulin and the peptides were proved to activate the IR signaling pathway [43]. Many previous studies have proved that *M. charantia* contains many peptides which are involved in the lowering of blood glucose level, and these studies support our findings [44].

Similarly, molecular docking of thirty-seven peptides was also performed against SGLT1 and the top five peptides were selected as potential ligands based on their interactions with active amino acids on the basis of their S-scores. From the docking analysis, Asn267, Tyr138, Tyr263, Ser368, and Thr431 were found to be active amino acids in the binding patterns with SGLT1. Previously, the interaction with Ser368 has been reported, and in the present study, Ser368 is repeatedly and actively involved in the binding patterns between receptor and selected ligands [45, 46]. Similarly, the SGLT1 inhibitor LX4211 ((2S,3R,4R,5S,6R)-2-(4-chloro-3-(4-ethoxybenzyl)phenyl)-6-(methylthio)tetrahydro-2H-pyran-3,4,5-triol) has been found to be involved in the inhibition of SGLT1 and consequently reduces the glucose absorption from the intestine. The results of these studies are in accordance with current findings. A number of antidiabetic peptides also have been reported from soy protein [47]. A great number of bioactive compounds from different parts of *M. charantia* have been found effective to treat diabetes. Shivanagoudra et al. [48] isolated two compounds (i.e., momordicoside G and gentisic acid 5-O-*β*-D-xyloside) and docked against *α*-amylase and *α*-glucosidase as receptor proteins. The momordicoside G showed the highest inhibition of *α*-amylase (70.5%), and gentisic acid 5-O-*β*-D-xyloside showed the highest inhibition of *α*-glucosidase (56.4%). In another study [49], the docking analysis of the compound nerolidol from *M. charantia* showed the best binding interactions with the diabetic enzyme glucokinase which is responsible to cause diabetes in humans.

The effectiveness and safety are the primary objectives for hunting a new drug as all drugs can help to combat diseases as well as cause harmful effects [50]. *In silico* analysis has played an increasingly significant part in the drug research and discovery by providing an effective way to assess multiple pharmacokinetics properties [51]. In this study, by taking into account all drug-like characteristics, only eight peptides were shortlisted (i.e., LIVA, TSEP, EKAI, LKHA, EALF, VAEK, DFGAS, and EPGGGG) followed by Lipinski's rule of five (Ro5). The peptide EPGGGG showed good binding affinity and efficacy for both IR and SGLT1 receptors. Some of the remaining ligands did not fulfill the drug-like criteria as they violated two or more rules of Ro5. The eight selected ligands also passed the evaluation through ADMET drug profiling to predict the capability or incapability of these peptides as potential drugs DM.

Among the parameters predicted by admetSAR, the Log*P* value tells about the permeability of a compound through a lipid membrane, and for a potential drug, it should be ≤5. The number of hydrogen bond acceptors and hydrogen bond donors of a drug describes its ability to bind with other compound(s) that consequently define its solubility and permeability. Nonrotatable bonds explain the molecular flexibility and permeation rate of a drug candidate. CYP450 is a superfamily of heme-containing enzymes that facilitate the metabolism of drugs. There are five different isoforms of CYP450 (CYP 3A4, 2D6, 1A2, 2C9, and 2C19). The oxidation of a drug is necessary for its proper functioning and excretion from the body, and for this reason, the consideration of action of drugs against these enzymes is necessary [52]. All the top eight ligands shortlisted in this study are noninhibitors of CYP50 family enzymes which is good for their metabolism. The Ames test predicts whether a selected ligand causes DNA mutation(s) or not. All the top eight peptides were predicted as non-Ames toxic. Structural alerts (SAs) are the main components which are the mastermind behind certain toxicities and carcinogenic behaviors. For the first time, it was reported that there is strong connection between some structural alters and chemical mutagenicity in *Salmonella* sp. [53]. Therefore, admetSAR predicts toxicity and carcinogenicity of ligands based on SAs. In our current findings, all top eight ligands are non-Ames toxic and noncarcinogenic and, therefore, they are safe and tolerable. Finally, all the eight peptides were predicted as having potential drug-like characters by successfully fulfilling the ADMET profiling criteria. The selected ligands have good affinity for receptors and efficacy to active receptors and acting as agonists of IR and inhibitors of SGLT1, DPP-IV, and GLUT2.

The foremost goal of the current study was to target those proteins that act as receptors in the regulation of glucose level in the body. Any insertion, deletion, and/or substitution in the amino acid sequence of these receptor proteins can lead to a deleterious effect in the maintenance of glucose level. Currently, many antidiabetic drugs have been excessively used but their disastrous outcomes make them undesirable and unsafe to use. This alarming situation requires the discovery of antihyperglycemic compounds with minimal side effects and maximal efficacy, and therefore, in the current study, we have explored natural peptides with great affinity for receptors involved in glucose regulation. *In silico* drug discovery is expected hunting the drugs quicker, cheaper, and more effective, but in spite of all these pros, the computational biology techniques have some limitations as various tools give different results for the same analyses, and therefore, one cannot fully rely on the results without wet lab investigation and validation [54].

## 5. Conclusion

Diabetes mellitus is an inevitable disorder, and in spite of all the available treatments, its consequences are rapidly enhancing epidemiologically. Protein-ligand docking and simulation approaches have greatly accelerated the discovery of novel antidiabetic agents. In the current study, using an in silico approach, we have discovered, designed, and proposed novel antidiabetic peptides for oral administration. Upon investigating dynamics stability, it was found that both complexes (i.e., LIVA-IR and DFGAS-SGLT1) were revealed to be stable as the RMSD values of both complexes were in good acceptable range. These ligands might reduce dependency on painful subcutaneous administration of insulin and on other drugs with a number of side effects. Out of thirty-seven peptides, the peptides LIVA, TSEP, EKAI, LKHA, EALF, VAEK, DFGAS, and EPGGGG were found to be the best ones as potential antidiabetic agents based on their interaction studies through molecular docking. These ligands were strictly evaluated through Lipinski's rule of five and ADMET profiling which strongly supported their antihyperglycemic properties, and therefore, these natural bioactive compounds would act as agonist of IR and inhibitors of SGLT1, DPP-IV, and GLUT2 and may lead to design potential drugs to combat diabetes with fewer or no side effects. A wet lab procedure has to be performed to further evaluate their activity as antidiabetic agents.

## Figures and Tables

**Table 1:** Property profile of selected peptides against five selected receptor proteins.

Sr. No.	Peptide	Receptor	S-score	RMSD	Interacting amino acids
1	KDDGHL	IR	-18.56	2.75	Ser1270, Asp1143, Glu1108, Glu1115, His1057
2	EPGGGG	IR	-16.71	1.85	Arg B1026. Glu A186, Asp B1343, His B1057, Lys B1147, Ala B1050, Ser A187
3	TSEP	IR	-15.66	1.21	Asp1143, Thr1145, Glu1115, Arg1101, Glu1108
4	VAEK	IR	-15.53	2.29	Glu1115, His1058, Ser1270, Asp1143, Phe1144, Glu1108
5	LIVA	IR	-14.00	1.73	Thr A178, His B1057, Cys B1056, Val B1059, Glu B1077, Lys B1147
6	ESIRD	SGLT1	-23.81	2.86	Thr431, ser368, Gln428, Asn142, Ser364, Ser66, Lys294, Gln69, Glu88
7	DSRHR	SGLT1	-23.64	2.46	Thr431, Ser368. Gln428. Ser91, Lys294, Ser365, Gln268, Asn267, Asn64, Ser66
8	RRKKV	SGLT1	-20.64	2.92	Thr431, Asn260, Asn267, Tyr263, Asn64, Ser435
9	PTRHM	SGLT1	-19.60	1.83	Tyr263, Gln68, Ala63, Ser365
10	DFGAS	SGLT1	-9.3259	1.5980	Asp189, Tyr176
11	PTRHM	DPP-IV	-10.1067	2.5395	Gln268, Thr375, Asn371
12	RRKKV	DPP-IV	-9.9189	1.7598	Val185, Asn267, Tyr269
13	KDDGHL	DPP-IV	-9.4991	1.4528	Ser368, Asn267, Val185
14	RSIHEP	DPP-IV	-9.0877	1.7136	Asn371, Val185
15	VAEK	DPP-IV	-8.1677	2.3228	Ser59, Ala63, Ser368
16	RRKKV	GLUT2	-10.5970	1.3552	Met174, Glu361, Arg432
17	RSIHEP	GLUT2	-10.5171	2.4307	Met174, Glu361
18	ERFDSG	GLUT2	-9.6986	1.7398	Gln429, Phe421
19	KDDGHL	GLUT2	-9.5645	1.5598	Glu282
20	PTRHM	GLUT2	-9.2187	1.4949	Arg432

**Table 2:** MM-GBSA binding free energy estimation (all the values are described in kcal/mol).

Energy component	LIVA-Insulin	DFGAS-SGLT1
van der Waals	-66.15	-50.27
Electrostatic	-52.45	-45.12
Polar solvation	35.69	39.41
Nonpolar solvation	-10.47	-6.87
Net gas phase	-118.6	-95.39
Net solvation	25.22	32.54
Net complex energy	-93.38	-62.85

**Table 3:** Net MM-GBSA binding free energy decomposition into residues of the receptors. The values are provided in kcal/mol.

Residue	LIVA-Insulin	Residue	DFGAS-SGLT1
Gln1004	-2.54	Ala62	-1.51
Gly1005	-1..85	Ala63	0.47
Ser1006	1.58	Asn64	1.47
Phe1007	-1.64	Ile65	1.02
Lys1085	-1.36	Ser66	-2.48
Ser1086	-1.00	Gly68	-1.35
Asp1083	-3.45	Tyr138	-1.23
His1130	0.25	Tyr176	-1.00
Asp1132	-0.89	Val185	-1.85
Arg1136	-1.65	Asp189	-2.58
Asn1137	0.05	Tyr263	-1.55
Met1139	1.63	Asn267	-1.27
Asp1150	-2.54	Gln268	-1.20
Phe1151	-1.02	Tyr269	-2.04
Gly1152	-1.50	Ile270	-0.87
Met1153	-2.54	Thr274	0.68
		Ser365	0.12
		Ser364	-1.08
		Ala367	-1.36
		Met369	-1.54
		Asn371	1.02

**Table 4:** Pharmacokinetic parameters important for bioavailability of compound drug-likeness properties of selected peptides.

Peptides	Target		Molecular properties^†^					
		MW	HBD	HBA	nrotb	Log*P*	*A*	Violations
LIVA	IR	414.54	5	6	12	0.51	111.74	0
TSEP	IR	432.43	7	10	14	-3.21	103.31	1
EKAI	IR	459.54	7	7	16	-1.09	116.22	1
LKHA	IR	467.57	7	7	15	-0.99	121.46	1
EALF	IR	478.55	6	6	14	0.03	123.58	1
VAEK	IR/DPP-IV	445.51	7	7	15	-1.48	111.41	1
DFGAS	IR/SGLT1/DPP-IV	495.48	8	8	15	-3.30	118.13	1
EPGGGG	IR/SGLT1	472.45	7	10	18	-3.34	111.79	1
GDVEC	SGLT1	521.55	9	9	16	-3.11	121.41	2
DDPTG	SGLT1	503.47	8	9	17	-4.19	116.53	2
PTRHM	DPP-IV/GLUT2	640.77	11	10	19	-2.87	165.54	3
RRKKV	DPP-IV/GLUT2	685.88	14	10	26	-3.22	181.62	3
DTDEL	DPP-IV	591.57	10	10	19	-3.42	135.64	3

^†^Molecular properties were calculated using SwissADME, an online tool. MW: molecular weight; HBD: number of hydrogen bond donors; HBA: number of hydrogen bond acceptors; nrotb: number of rotatable bonds; Log*P*: the logarithm of octanol/water partition coefficient; *A*: molar refractivity.

**Table 5:** ADMET profiling enlisting absorption, metabolism, and toxicity-related drug-like parameters of best selected peptides.

	Peptides							
	LIVA	TSEP	EKAI	LKHA	EALF	VAEK	DFGAS	EPGGGG
*Absorption*								
BBB	+	+	+	+	–	+	+	+
HIA	–	–	–	+	+	–	–	+
Caco-2 permeability	Caco-2-	Caco-2-	Caco-2-	Caco-2-	Caco-2-	Caco-2-	Caco-2-	Caco-2-
PGS	Substrate	NS	Substrate	Substrate	Substrate	NS	NS	Substrate
PGI	NI	NI	NI	NI	NI	NI	NI	NI
ROCT	NI	NI	NI	NI	NI	NI	NI	NI

*Metabolism*								
CYP3A4 substrate	Substrate	NS	Substrate	Substrate	NS	NS	NS	Substrate
CYP2C9 substrate	NS	NS	Substrate	NS	NS	NS	NS	Substrate
CYP2D6 substrate	NS	NS	NS	NS	NS	NS	NS	NS
CYP3A4 inhibition	NI	NI	NI	NI	NI	NI	NI	NI
CYP2C9 inhibition	NI	NI	NI	NI	NI	NI	NI	NI
CYP2C19 inhibition	NI	NI	NI	NI	NI	NI	NI	NI
CYP2D6 inhibition	NI	NI	NI	NI	NI	NI	NI	NI
CYP1A2 inhibition	NI	NI	NI	NI	NI	NI	NI	NI

*Toxicity*								
Ames toxicity	NAT	NAT	NAT	NAT	NAT	NAT	NAT	NAT
Carcinogens	NC	NC	NC	NC	NC	NC	NC	NC

BBB: blood-brain barrier; HIA: human intestinal absorption; PGS: P-glycoprotein substrate; PGI: P-glycoprotein inhibitor; ROCT: renal organic cation transporter; NS: nonsubstrate; NI: noninhibitor; NAT: non-Ames toxic; NC: noncarcinogenic.

## Data Availability

The data used to support the findings of this study are available from the corresponding author upon request.

## Supplementary Materials

Supplementary Materials Figure S1: interactions (a) and binding pattern (b) of chaetochromin with IR as a positive control. Figures S2–S4: interactions and binding patterns of EPGGGG, TSEP, and VAEK peptides with IR. Figures S5–S7: interactions and binding patterns of DSRHR, RRKKV, and PTRHM peptides with SGLT1 receptor. Figure S8: interactions (a) and binding patterns (b) of phlorizin with SGLT1 receptor as a positive control. Figures S9–S11: interactions and binding patterns of PTRHM, RRKKV, and KDDGHL peptides with DPP-IV receptor. Figures S12–S14: interactions and binding patterns of RRKKV, RSIHEP, and ERFDSG peptides with GLUT2 receptor. Figure S15: SASA analysis of the systems.
