# Supplementary material for: Molecular Docking and Simulation Studies of Antidiabetic Agents Devised from Hypoglycemic Polypeptide-P of Momordica charantia
Source: Biomed Res Int. 2021 Sep 17;2021:5561129. doi: 10.1155/2021/5561129 (PMC8476269; doi:10.1155/2021/5561129)
Supplement: Supplementary Materials — Figure S1: interactions (a) and binding pattern (b) of chaetochromin with IR as a positive control. Figures S2–S4: interactions and binding patterns of EPGGGG, TSEP, and VAEK peptides with IR. Figures S5–S7: interactions and binding patterns of DSRHR, RRKKV, and PTRHM peptides with SGLT1 receptor. Figure S8: interactions (a) and binding patterns (b) of phlorizin with SGLT1 receptor as a positive control. Figures S9–S11: interactions and binding patterns of PTRHM, RRKKV, and KDDGHL peptides with DPP-IV receptor. Figures S12–S14: interactions and binding patterns of RRKKV, RSIHEP, and ERFDSG peptides with GLUT2 receptor. Figure S15: SASA analysis of the systems. [file 5561129.f1.docx]

**Molecular docking and simulation studies of antidiabetic agents devised from hypoglycemic polypeptide-P of *Momordica charantia***

***Supplementary Materials***

| **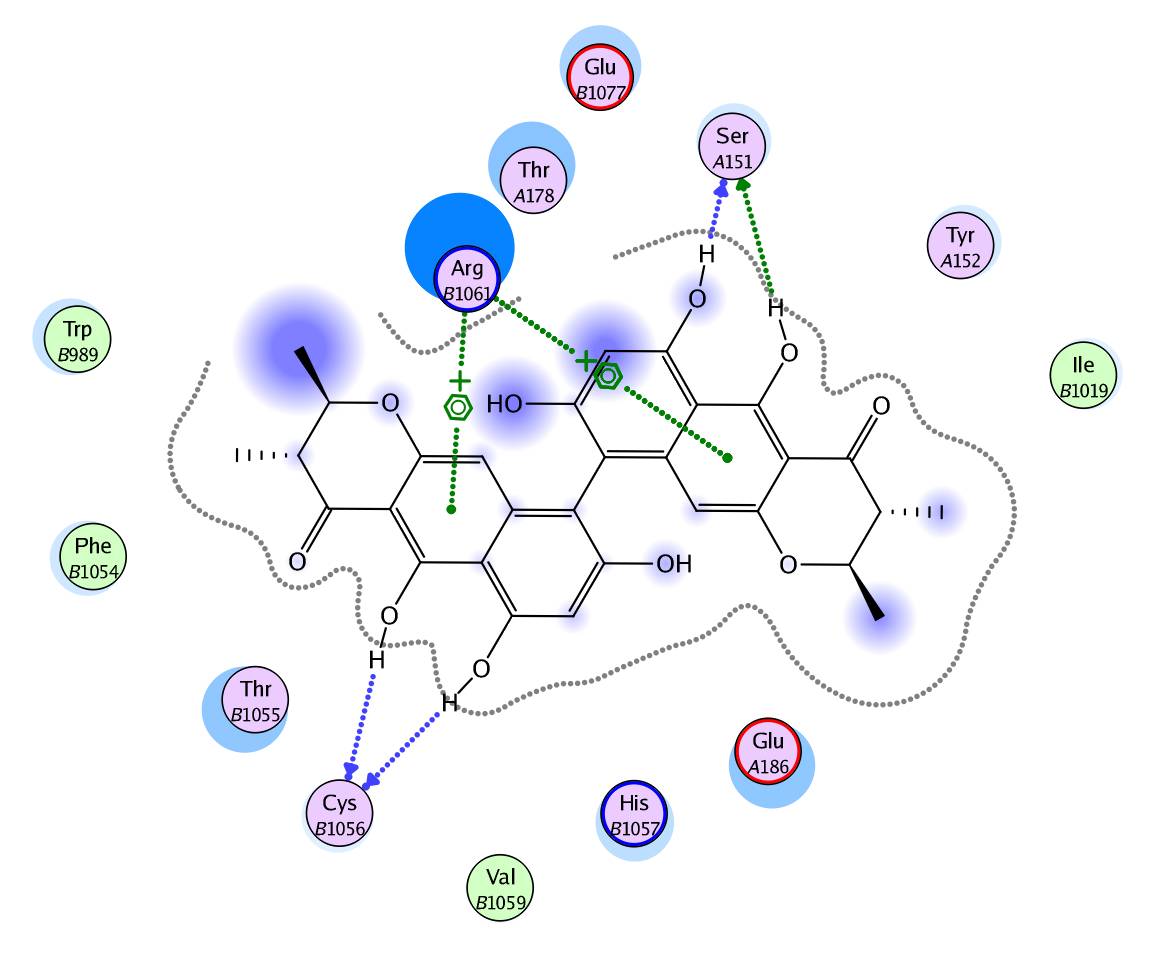**  **(a)** | **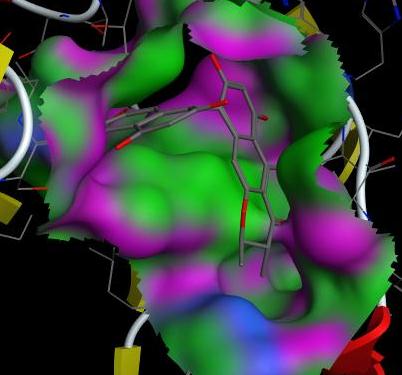**  **(b)** |
| --- | --- |

FIGURE S1: Interactions (a) and binding pattern (b) of chaetochromin with insulin receptor as a positive control

| 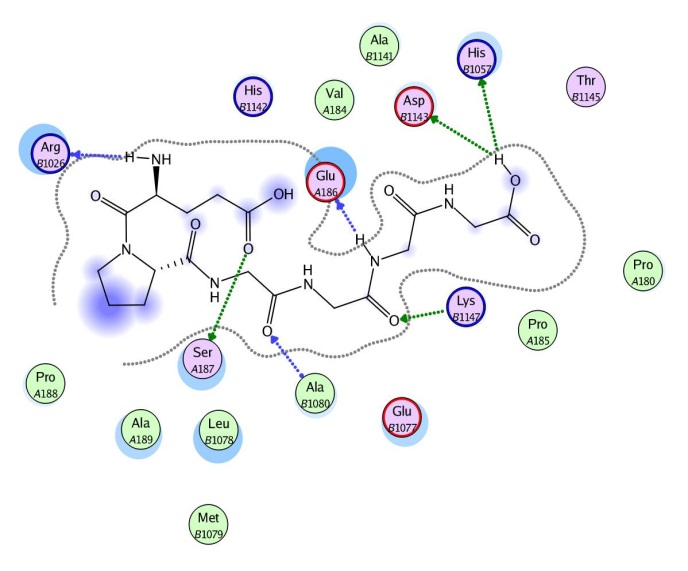  **(a)** | 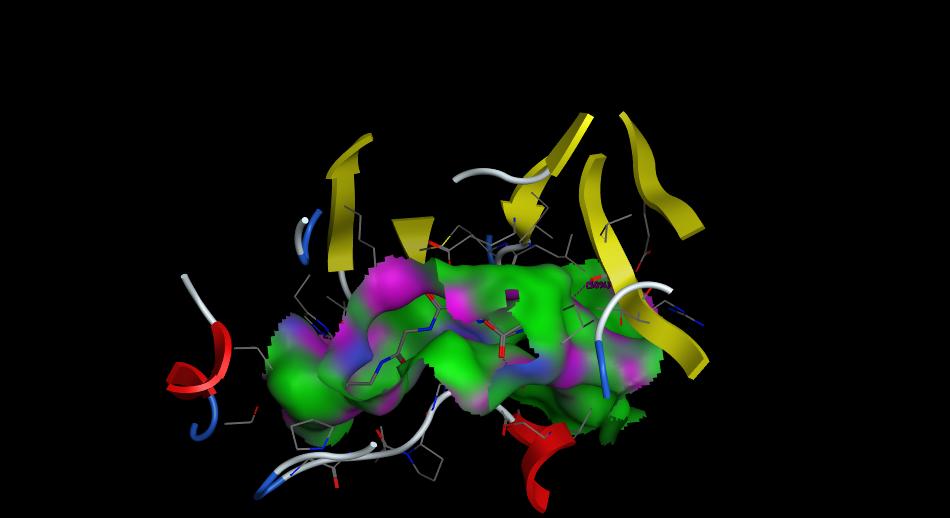  **(b)** |
| --- | --- |

FIGURE S2: Interaction (a) and binding patterns (b) of EPGGGG peptide with insulin receptor

| 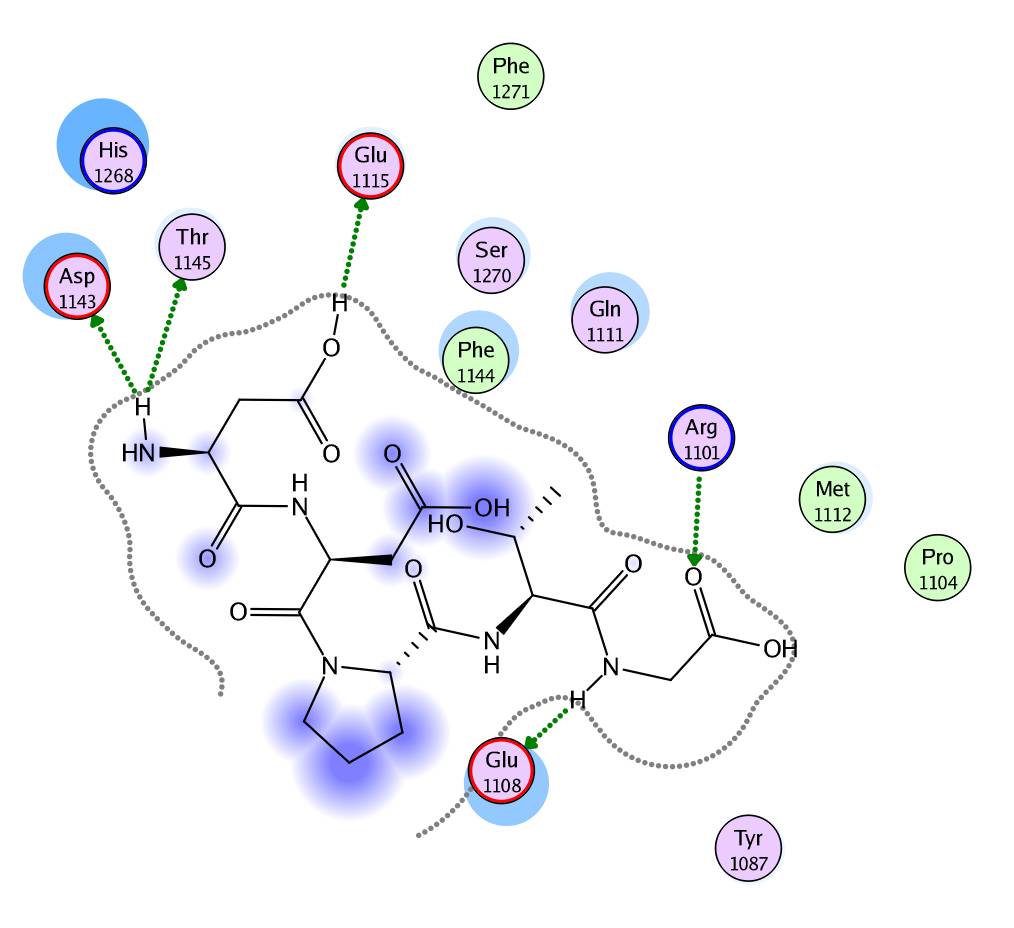  **(a)** | 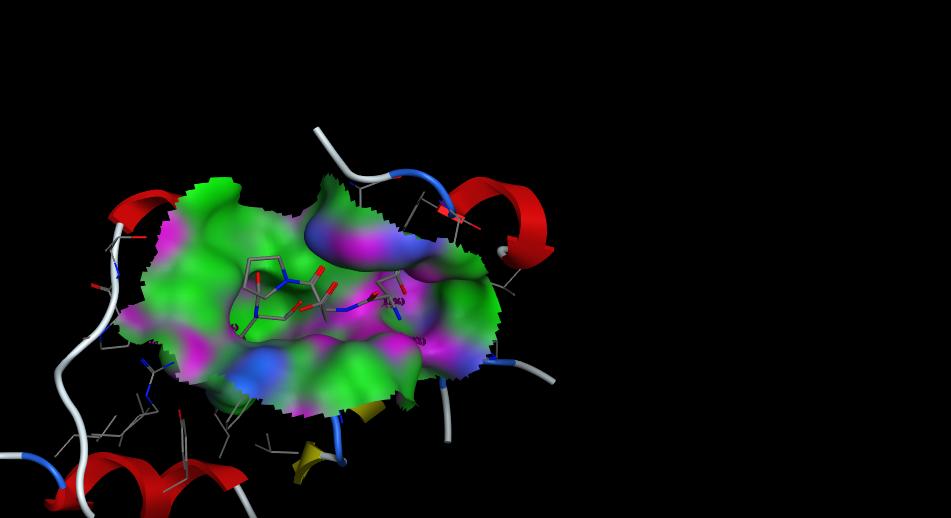  **(b)** |
| --- | --- |

FIGURE S3: Interaction (a) and binding patterns (b) of TSEP peptide with insulin receptor

| 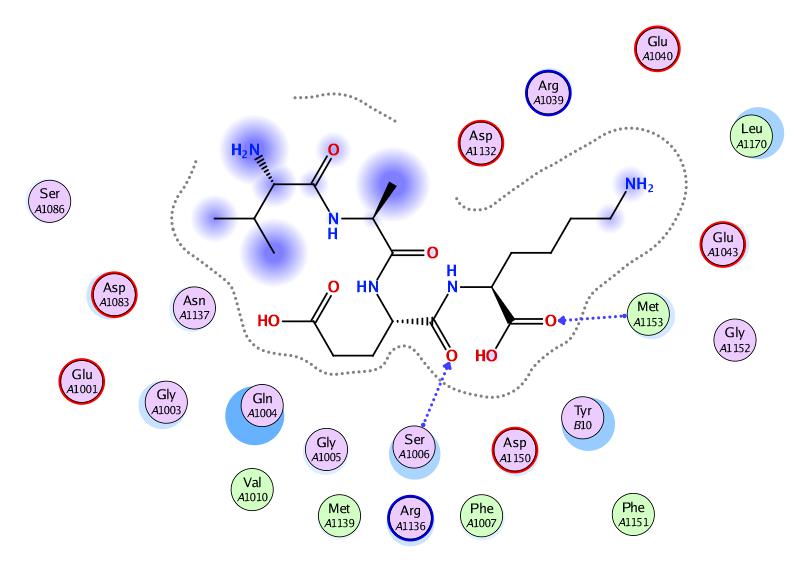  **(a)** | 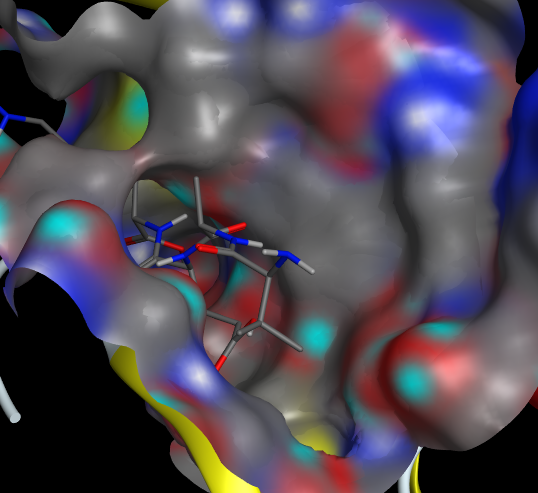  **(b)** |
| --- | --- |

FIGURE S4: Interaction (a) and binding patterns (b) of VAEK peptide with insulin receptor

| 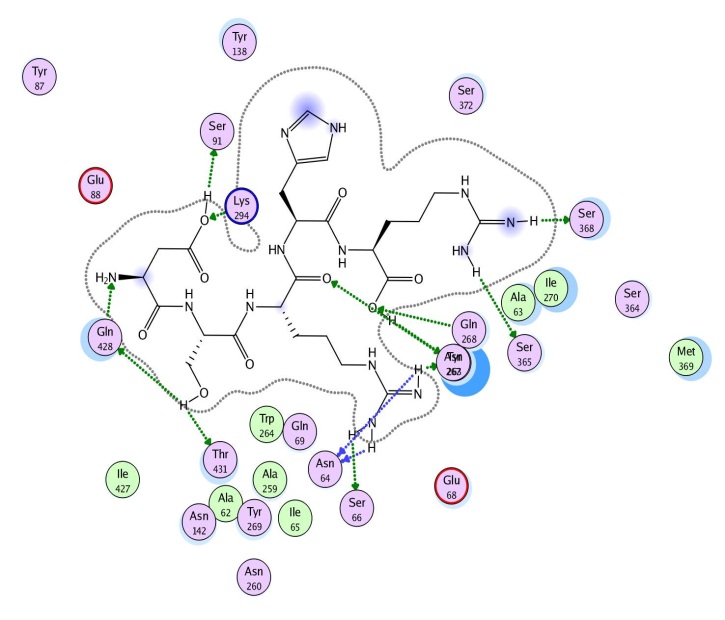  **(a)** | 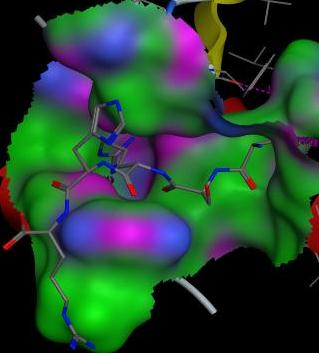  **(b)** |
| --- | --- |

FIGURE S5: Interaction (a) and binding patterns (b) of DSRHR peptide with SGLT1 receptor

| 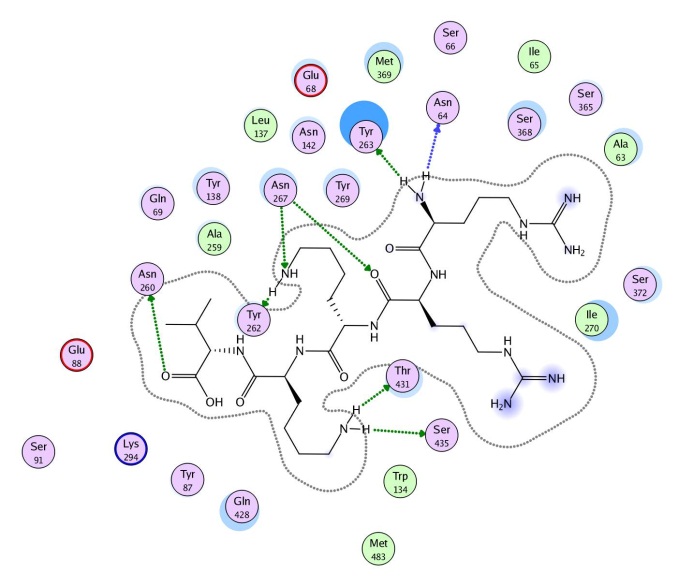  **(a)** | 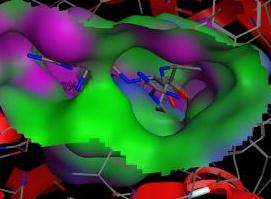  **(b)** |
| --- | --- |

FIGURE S6: Interaction (a) and binding patterns (b) of RRKKV peptide with SGLT1 receptor

| 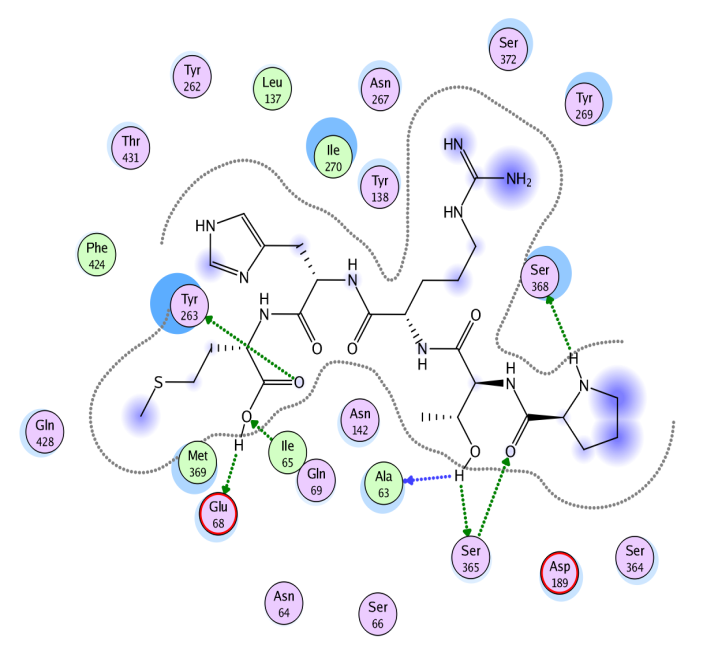  **(a)** | 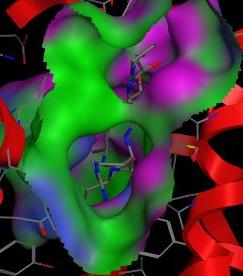  **(b)** |
| --- | --- |

FIGURE S7: Interaction (a) and binding patterns (b) of PTRHM peptide with SGLT1 receptor

| 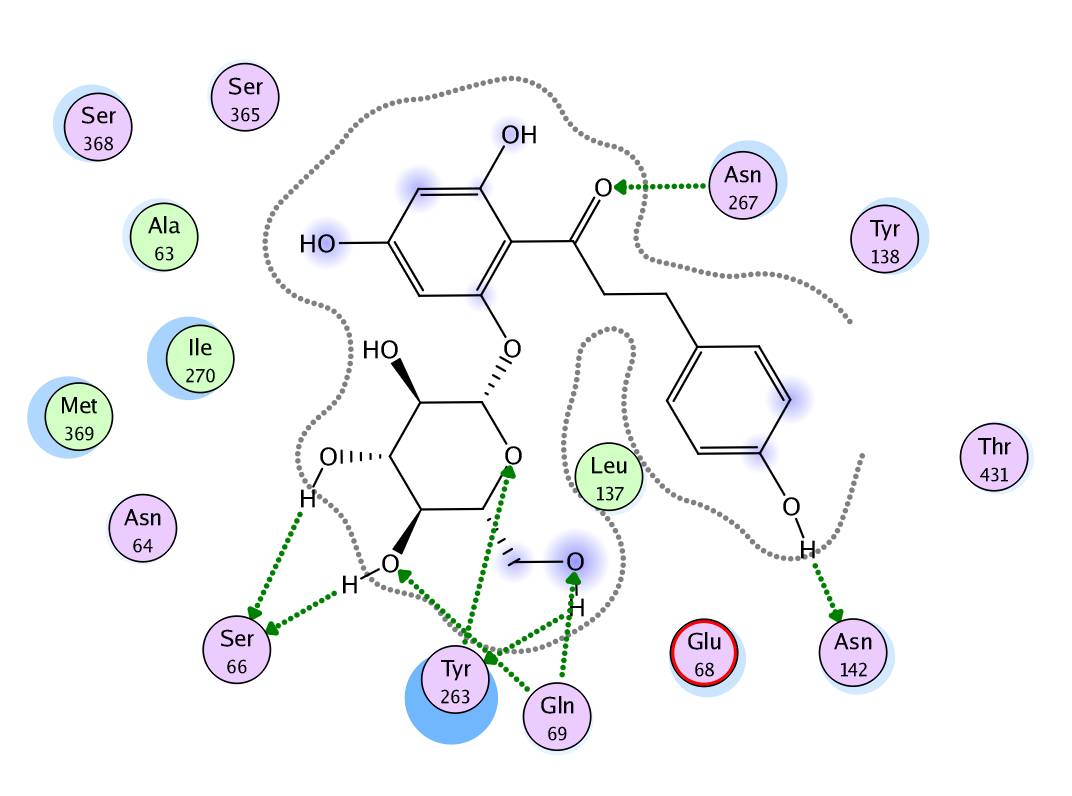  **(a)** | 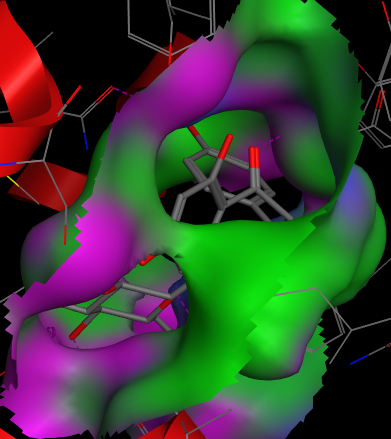  **(b)** |
| --- | --- |

FIGURE S8: Interaction (a) and binding patterns (b) of phlorizin with SGLT1 receptor as a positive control

| 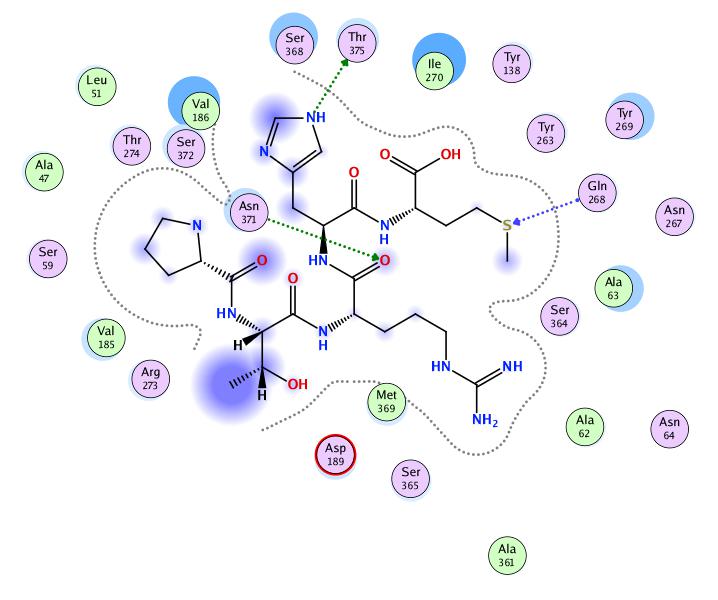  **(a)** | 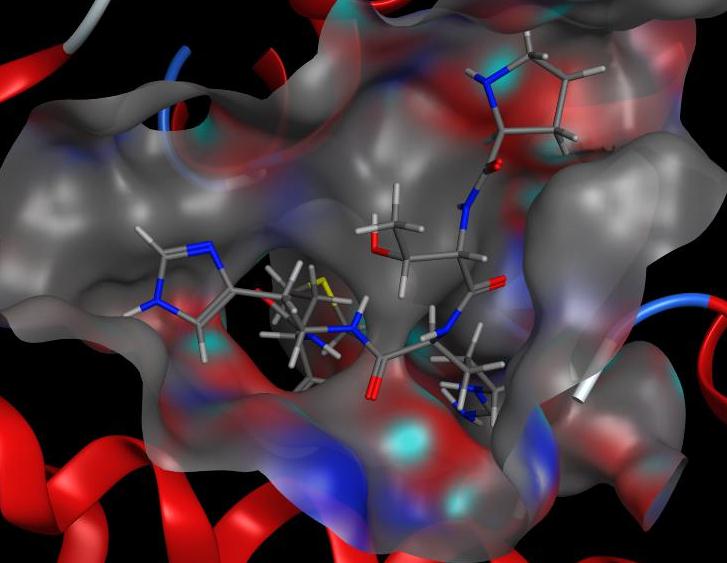  **(b)** |
| --- | --- |

FIGURE S9: Interaction (a) and binding patterns (b) of PTRHM with DPP-IV receptor

| 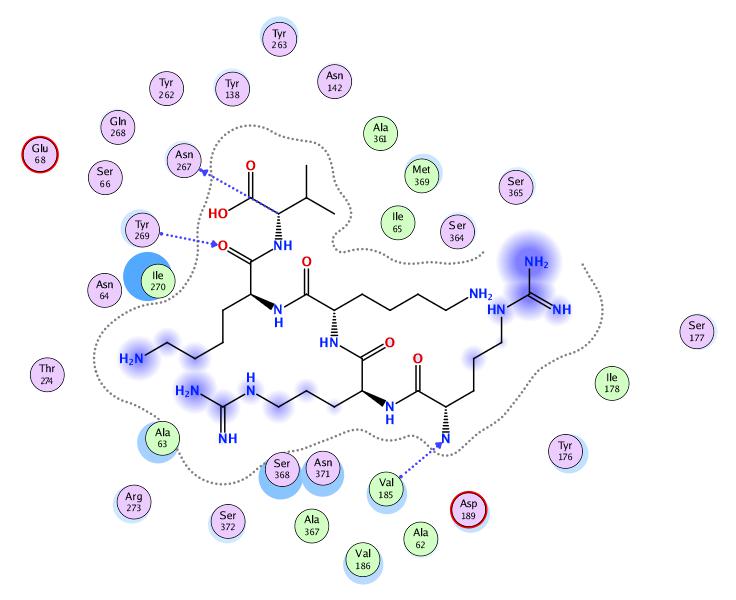  **(a)** | 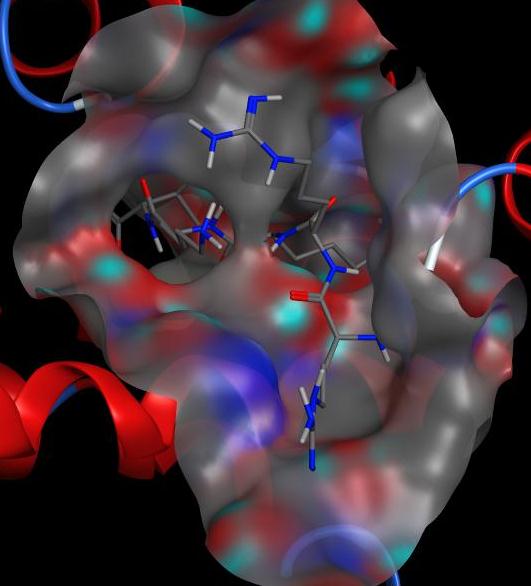  **(b)** |
| --- | --- |

FIGURE S10: Interaction (a) and binding patterns (b) of RRKKV with DPP-IV receptor

| 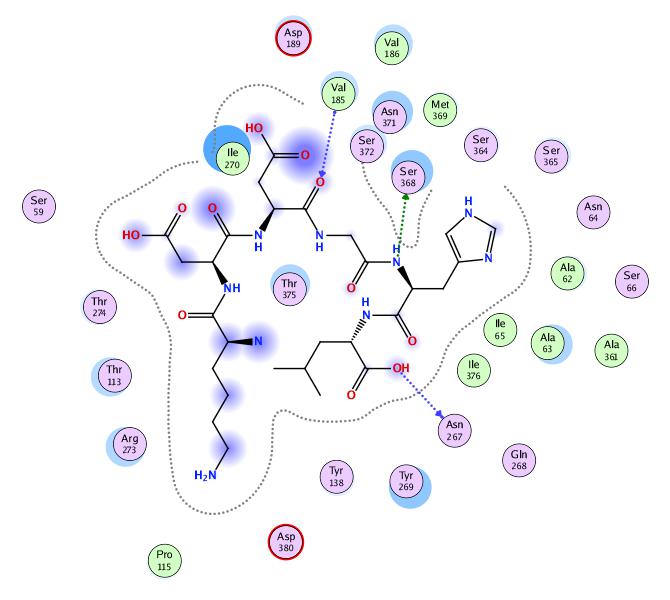  **(a)** | 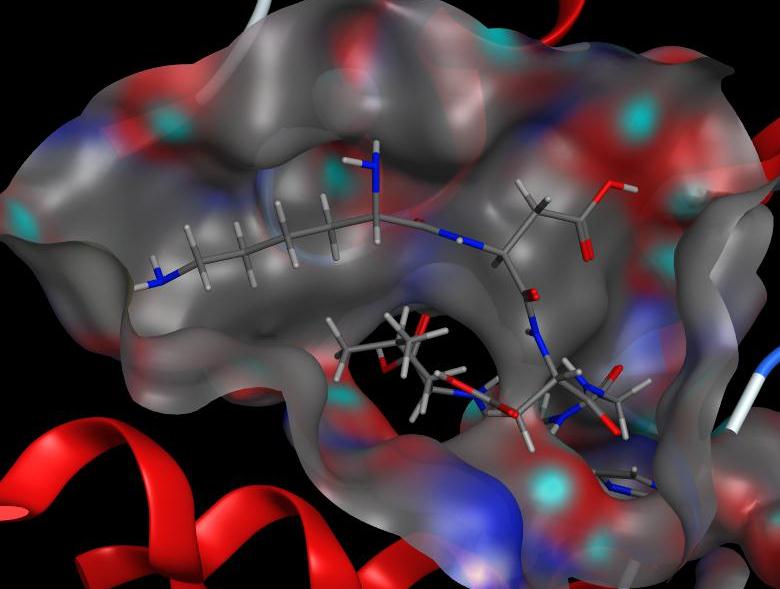  **(b)** |
| --- | --- |

FIGURE S11: Interaction (a) and binding patterns (b) of KDDGHL with DPP-IV receptor

| 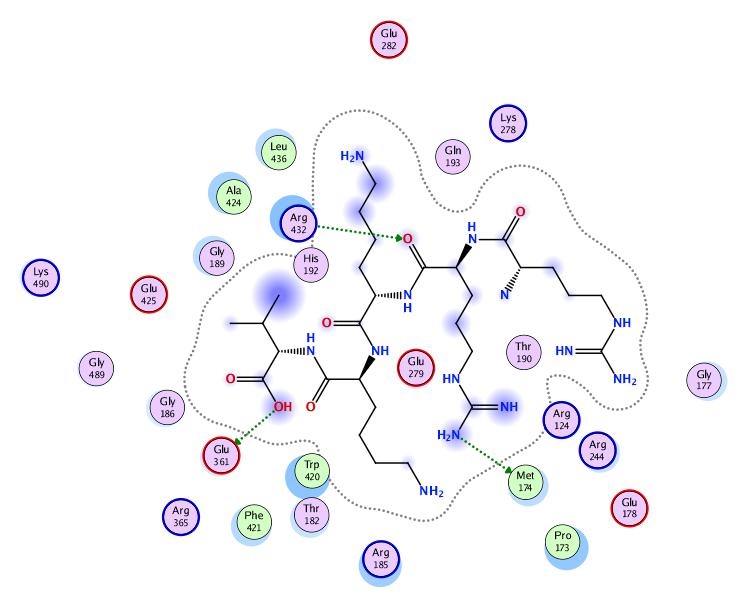  **(a)** | 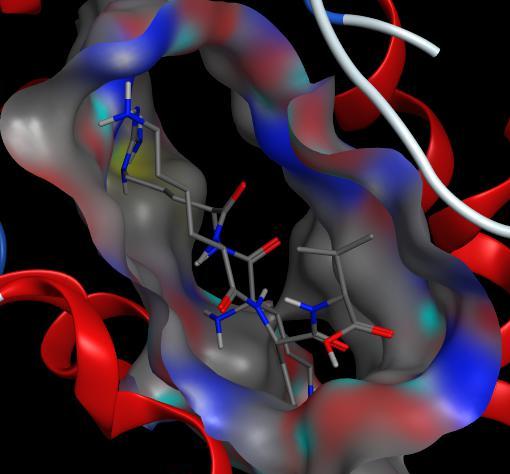  **(b)** |
| --- | --- |

FIGURE S12: Interaction (a) and binding patterns (b) of RRKKV with GLUT2 receptor

| 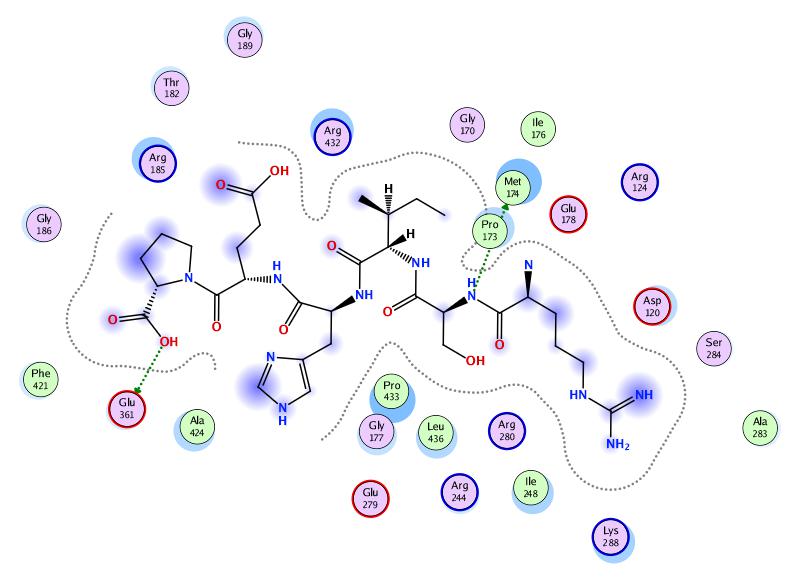  **(a)** | 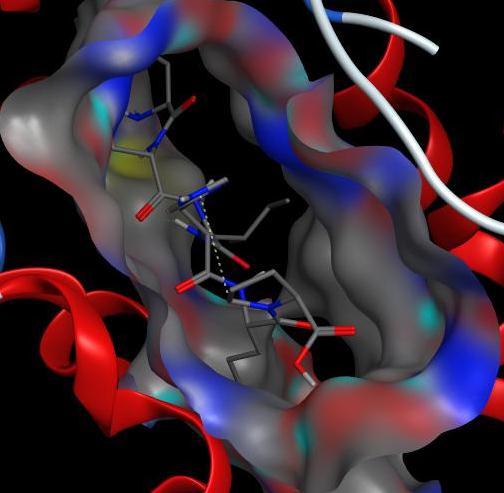  **(b)** |
| --- | --- |

FIGURE S13: Interaction (a) and binding patterns (b) of RSIHEP with GLUT2 receptor

| 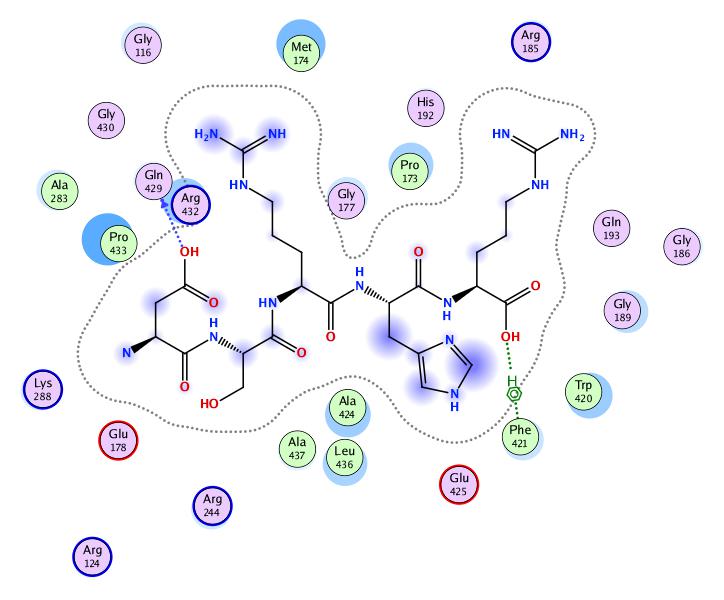  **(a)** | 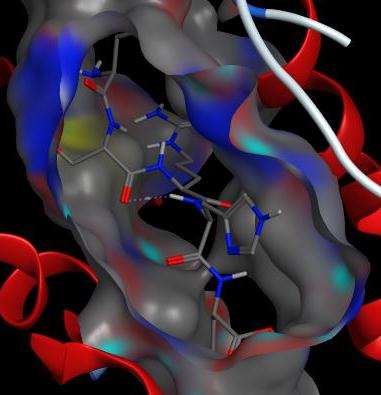  **(b)** |
| --- | --- |

FIGURE S14: Interaction (a) and binding patterns (b) of ERFDSG with GLUT2 receptor

**
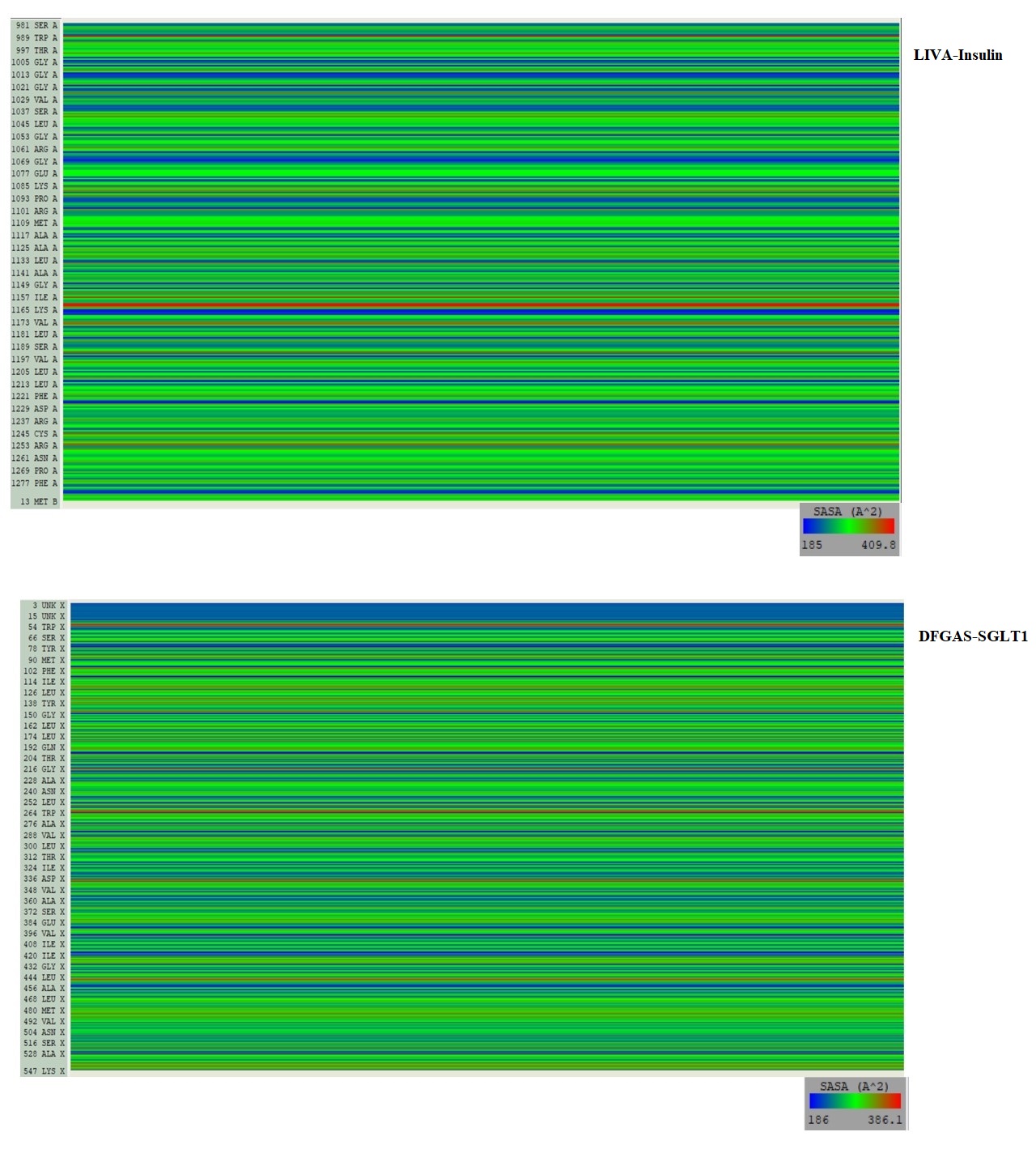
**

FIGURE S15: SASA analysis of the systems
